# Pathway-Based Analysis of a Melanoma Genome-Wide Association Study: Analysis of Genes Related to Tumour-Immunosuppression

**DOI:** 10.1371/journal.pone.0029451

**Published:** 2011-12-27

**Authors:** Nils Schoof, Mark M. Iles, D. Timothy Bishop, Julia A. Newton-Bishop, Jennifer H. Barrett, GenoMEL consortium

**Affiliations:** 1 Section of Epidemiology and Biostatistics, Leeds Institute of Molecular Medicine, University of Leeds, Leeds, United Kingdom; 2 Department of Medical Epidemiology and Biostatistics, Karolinska Institute, Stockholm, Sweden; 3 The Melanoma Genetics Consortium (GenoMEL); University of Michigan, United States of America

## Abstract

Systemic immunosuppression is a risk factor for melanoma, and sunburn-induced immunosuppression is thought to be causal. Genes in immunosuppression pathways are therefore candidate melanoma-susceptibility genes. If variants within these genes individually have a small effect on disease risk, the association may be undetected in genome-wide association (GWA) studies due to low power to reach a high significance level. Pathway-based approaches have been suggested as a method of incorporating *a priori* knowledge into the analysis of GWA studies. In this study, the association of 1113 single nucleotide polymorphisms (SNPs) in 43 genes (39 genomic regions) related to immunosuppression have been analysed using a gene-set approach in 1539 melanoma cases and 3917 controls from the GenoMEL consortium GWA study. The association between melanoma susceptibility and the whole set of tumour-immunosuppression genes, and also predefined functional subgroups of genes, was considered. The analysis was based on a measure formed by summing the evidence from the most significant SNP in each gene, and significance was evaluated empirically by case-control label permutation. An association was found between melanoma and the complete set of genes (p_emp_ = 0.002), as well as the subgroups related to the generation of tolerogenic dendritic cells (p_emp_ = 0.006) and secretion of suppressive factors (p_emp_ = 0.0004), thus providing preliminary evidence of involvement of tumour-immunosuppression gene polymorphisms in melanoma susceptibility. The analysis was repeated on a second phase of the GenoMEL study, which showed no evidence of an association. As one of the first attempts to replicate a pathway-level association, our results suggest that low power and heterogeneity may present challenges.

## Introduction

The incidence of melanoma is increased in chronically immunosuppressed individuals, such as recipients of transplanted organs, indicating that the immune system restricts the outgrowth of melanoma cells [1], [2]. Anti-melanoma immune responses seem to be possible, but certain mechanisms probably at the tumour site circumvent these and give rise to tumour development [3]. Furthermore a potent risk factor for melanoma is sunburn [4], and seminal animal studies established that ultraviolet-induced local and systemic immunosuppression is important in the pathogenesis of melanoma. The hypothesis is that intense sun exposure induces both genetic changes, resulting in tumour antigenicity, and an inability of the immune system to detect those changes [5].

Within the concept of tumour immunosurveillance [6], transformed cells are recognized by antigen-presenting cells (APCs) (essentially dendritic cells (DCs)), and the latter differentiate into activated states. The activated APCs further interact with tumour-specific T helper lymphocytes and induce their activation, which in turn leads to activation of tumour-specific cytotoxic T lymphocytes (cTLs). These cTLs recognise the nascent tumour cells and induce their elimination. Many molecular mechanisms are known to influence immunological capacity. The DCs are known to exist in a state that induces immune tolerance and in an activated state, which induces immunity [7]. It has been shown that melanoma appears to induce tolerogenic DCs (tDCs) capable of inducing immunosuppression [8]. Two major mechanisms are known to prevent T lymphocyte activation and resulting immune responses. Firstly, T lymphocytes can differentiate into a state of anergy indicating their functional inactivation [9]. The analysis of the microenvironment around melanoma cells showed the presence of anergic T-cells, and these might also contribute to the lack of anti-tumoral immune responses [10], [11]. Secondly, regulatory T lymphocytes (Treg) have strong immunosuppressive properties through multiple modes of action [12]. Treg cells have also been found in melanoma lesions and could induce immunotolerance [13], [14]. The interaction between tumour cells, APCs and T lymphocytes and their respective effects are strongly dependent on molecules on the surface of each cell. The significance of one of these, the cytotoxic T-lymphocyte-associated protein 4 (*CTLA4*), is indicated by the recent encouraging clinical trials using antibodies directed to this immunosuppressive costimulatory molecule in patients with stage IV melanoma [15]. One major effector function of these immunosuppressive cell types is the secretion of factors with immune regulatory functions. However, the tumour cells are also capable of secretion of these factors and may thereby recruit (e.g. by chemokine (C-C motif) ligand 17/22 (*CCL17/22*)) or lead to the differentiation of immunosuppressive cell types (e.g. by indolamine-2,3-dioxygenase 1 (*IDO1*) or Interleukin 10 (*IL10*)) [16].

Several candidate gene studies have been reported focussing on variants within genes related to these immunosuppressive mechanisms [17]–[20]. Most of these studies analysed only a limited number of single nucleotide polymorphisms (SNPs) or had relatively small sample sizes, and some of the results are inconsistent.

In a genome-wide association (GWA) study of melanoma carried out by the GenoMEL consortium, association was confirmed between disease susceptibility and variants related to melanocortin-1 receptor (*MC1R*) and tyrosinase (*TYR*), and a new locus at chromosome 9p21 was identified [21]. In GWA study analyses, usually each individual SNP is tested for association with the disease, and only loci approaching “genome-wide” statistical significance (e.g. p<5×10^−7^) are followed up. GWA studies are powerful at identifying risk variants, which are common in a population and have low to moderate penetrance. However, other loci in the large GWA data sets are also likely to be associated with disease risk but are indistinguishable from false positive results using this approach. Thus, candidate gene approaches and GWA studies have contributed to the understanding of genetic disease risk, but the latter are underpowered to detect weak associations with susceptibility to disease at the genome-wide significance level.

To overcome the limitations of these approaches, the analysis of functional gene sets, so-called pathway-based analysis, has recently been proposed [22]–[25]. The large data sets from GWA studies can be re-analysed incorporating *a priori* knowledge into the analysis in an attempt to identify new risk factors. The idea behind pathway-based GWA study analysis is that SNPs in a group of genes with a shared biological function may show significant association at the pathway level, even though no individual SNP shows association at a stringent level of statistical significance. Thus further information about disease aetiology may be obtained using the existing data from the GWA study. Recently, pathway-based approaches have been applied to GWA studies of several complex diseases [26]–[31]. Most analyses have taken an agnostic approach and included a comprehensive pathway search, using databases like Gene Ontology or the Kyoto Encyclopedia of Genes and Genomes. In this study, a candidate pathway analysis is applied to data from the GenoMEL melanoma GWA study [21]. Instead of an analysis of many pathways, the pathway of tumour-immunosuppression was selected, and a comprehensive analysis of SNPs in this pathway in relation to melanoma susceptibility was conducted.

## Materials and Methods

### Study design and subjects

This study reports a further statistical analysis of the first phase of the melanoma GWA study of the GenoMEL consortium, full details of which have been described elsewhere [21]. Briefly, participating GenoMEL groups (Barcelona, Brisbane, Emilia-Romagna, Genoa, Leiden, Leeds, Lund, Paris, Stockholm and Sydney) contributed 1650 melanoma cases with either a family history of melanoma (without CDKN2A mutations), early disease onset (age<40 years) or multiple primary sites to enrich for cases with greater genetic predisposition. Controls were provided by the same GenoMEL groups from the same populations, and an additional 2938 controls were contributed by the Centre National de Genotypage (CNG, France) and the Wellcome Trust Case Control Consortium (WTCCC, UK). The anonymised data are stored on a secure server, and personal information is held only by the contributing centre. Each participating group holds local ethical approval for the GWA analysis and written informed consent from the participants [21].

### Genotyping and sample exclusion

Genotyping was performed through ServiceXS (Netherlands) using the Illumina HumanHap300 Bead-Chip version 2 duo array and by CNG in Paris using the Illumina HumanCNV370k array. Additional French and WTCCC controls were genotyped on the Illumina HumanHap300 BeadChip version 2 duo array. In total, 1650 cases and 4336 controls were genotyped. Samples were excluded if (i) the overall call rate was less than 97%, (ii) there was evidence of non-European origin from principal components analysis (PCA), (iii) sex as inferred from genotyping did not match reported sex, or (iv) there was evidence of first-degree relationship or genetic identity with another sample (for detailed information see [21]). This quality control led to the exclusion of 111 cases and 419 controls, mainly due to a call rate (<97%) (predominantly from the group of additional French controls).

### Selection of genes and SNPs

In this study 43 genes, associated with the suppression of immune responses, were selected, based on an extensive literature review and blind to the results of the GWA study (Table 1). The genes were further divided into subgroups related to suppression by regulatory T-cells (*Treg*), the induction of T-lymphocyte anergy (*Anergy*), regulation by costimulatory receptors (*Costim*.), regulation by dysfunctional, tolerogenic dendritic cells (*tDC*), and the secretion of suppressive factors (*Secreted*). Some genes can be categorised into more than one subgroup, as indicated in Table 1. The chromosomal location of the genes was retrieved from the HapMap database (NCBI build 36) and 100 kilobase flanking regions were included. Overlapping genes were merged into one, leading to 39 genomic regions to be analysed. From within these regions, 1178 SNPs genotyped in this study were obtained using the dbSNP database (build 126).

**Table 1:** Selected genes included into the analysis and division into subgroups.

GeneSymbol	EntrezGeneID	Chromo-some	Treg	Anergy	Costim.	tDC	Secreted
BTLA	151888	3			X		
BTNL2	56244	6			X		
CBLB	868	3		X			
CCL17/CCL22	6361/6367	16	X				X
CD160	11126	1			X		
CD274/PDCD1LG2	29126/80380	9			X		
CD28/ICOS/CTLA4	940/29851/1493	2	X		X		
CD40	958	20			X		
CD40LG	959	X			X		
CD80	941	3			X		
CD86	942	3			X		
DGKA	1606	12		X			
FOXP3	50943	X	X				
ICOSLG	23308	21			X		
IDO1	3620	8	X			X	X
IL10	3586	1	X			X	X
Il10RA	3587	11	X			X	
IL10RB	3588	21	X			X	
IL12A	3592	3					X
IL12B	3593	5					X
IL12RB1	3594	19	X				
IL17A	3605	6	X				X
IL17RA	23765	22	X				
IL17RB	55540	3	X				
ITCH	83737	20		X			
LGALS1	3956	22					X
LGALS3	3958	14					X
LILRB2	10288	19				X	
LILRB4	11006	19				X	
PDCD1	5133	2			X		
RNF128	79589	X		X			
TGFB1	7040	19	X			X	X
TGFB2	7042	1	X			X	X
TGFBR1	7046	9	X			X	
TGFBR2	7048	3	X			X	
TGFBR3	7049	1	X			X	
TNFRSF18	8784	1	X				
TREML2	79865	6			X		
VDR	7421	12	X			X	

### SNP quality control

Quality control was based on the minor allele frequency, genotype call rate, exact Hardy-Weinberg equilibrium (HWE) test and differences in allele frequencies between six geographical regions of the participating GenoMEL centres (grouped as Sweden, Australia, Italy, United Kingdom/Netherlands, France and Spain) (based on a χ^2^ test with 5 degrees of freedom (d.f.)). 60 SNPs with a call rate below 97% were excluded from the analysis. Furthermore, 5 SNPs with an exact HWE p-value<10^−5^ and no regional differences in the allele frequencies (χ^2^ test p-value>0.001) were excluded.

### Pathway-based analysis

The association between susceptibility to melanoma and the immunosuppression gene set (and the respective subgroups (Table 1)) was analysed using an approach in which each gene (or genomic region) in the pathway is represented by the maximally associated SNP within the gene [25], [32]. First, logistic regression was performed for each SNP, assuming an additive genetic model and with the GenoMEL regional group (defined above) included as a covariate, using the PLINK software package [33]. Then, 5000 case-control label permuted data sets were analysed in the same way. These data sets were created by permuting case-control status within clusters (formed by the GenoMEL regional groups) in order to retain the original structure of the GWA study. All PLINK result files (based on observed and permuted data) were further analysed using the R software package [34]. The maximal z-value (i.e. the absolute value of the coefficient for the per-allele SNP effect, divided by its standard error) from the logistic regression analysis was assigned to each of the k genes, for the observed data set and each permuted data set. To evaluate the statistical significance of the gene sets, the SUMSTAT (Σ_k_|zi|) and SUMSQ = Σ_k_ z^2^) statistics were calculated for the observed and permuted gene sets [32]. An empirical p-value for the association of a gene set with melanoma susceptibility is calculated by the number of times the permuted gene statistic exceeds the original test statistic, divided by the number of permutations. This method thus provides a test of the null hypothesis that none of the genes is associated with melanoma risk.

Random gene sets were tested for association with melanoma using the same approach (Table 5 in Information S1). These gene sets were randomly selected from a list containing 18410 genes (Refseq sequences with status “mRNA” from HG18 downloaded from the HapMap database). For each random gene set, 43 genes were sampled, but several sets included genes containing no SNPs in the GenoMEL GWA data set (mainly from the X chromosome), reducing the number of genes to between 35 and 42 genes per set. With these 100 random gene sets the pathway analysis was conducted as described before.

## Results

Overall, the data for 1178 genotyped SNPs in the 39 selected genomic regions in 1539 melanoma cases and 3917 controls were available from the GenoMEL GWA study. 1113 SNPs remained after quality control and were used for the pathway-based analyses. The smallest nominal p-value from the logistic regression analysis was found for SNP rs873061 in the region of the lectin galactoside-binding soluble 3 (*LGALS3*) gene (p_logreg_ = 0.00033, odds ratio 0.84, 95% CI: 0.76–0.92). Thus no SNP showed association with risk of melanoma at a genome-wide significance level or after correcting for the number of SNPs in this study (Bonferroni correction with significance level of 0.05 corrected for 1113 tests).

Analysing the data using the pathway analysis based on the most significant SNP in each gene showed evidence of association of the complete set of immunosuppressive genes with the risk of melanoma (SUMSTAT p_emp_ = 0.002 from 5000 permutations, Table 2). Two subgroups of genes were primarily responsible for this result; the subgroup of secreted factors showed the strongest association (SUMSTAT p_emp_ = 0.0004), followed by the subgroup of genes associated with tDCs (SUMSTAT p_emp_ = 0.006). The results were very similar using the SUMSQ statistic instead of SUMSTAT for the pathway statistic. The three other groups (Treg, Anergy and Costim.) showed no significant results at the 5% level using either statistic.

**Table 2:** Pathway analysis for all genes and the gene subgroups.

Set	SUMSTAT	SUMSQ
All genes	*0.0020*	*0.0032*
Anergy	0.1378	0.1548
Costim.	0.1022	0.1556
Treg	0.0874	0.0812
Secreted	*0.0004*	*0.0004*
tDC	*0.0060*	*0.0082*

Empirical p-values established by 5000 in-cluster (GenoMEL regional group) label permutations are shown for the pathway statistics SUMSTAT and SUMSQ. Nominally significant results are shown in italics.

Several further analyses were conducted to test the validity of these results. First, these results remained stable when including the first three principal components (established to account for population stratification [21]) as covariates in the logistic regression (Table 1 in Information S1, p_emp_ = 0.006 from 1000 permutations). In particular, the subgroup of secreted immunosuppressive factors remained most significantly associated (p_emp_ = 0.002). Secondly, 100 random gene sets were tested, applying the same methodology (Table 5 in Information S1). Only 10 of the 100 random gene sets showed a nominally significant result (p<0.05) whichever test statistic was used (SUMSTAT or SUMSQ). Thirdly, the observed data set was replaced by a permuted data set and the complete procedure was repeated to test for any flaws in the programmed R algorithm (Table 2 in Information S1). In this analysis, there was no evidence of overall association, and only the subgroup of genes related to anergy reached nominally significant results (SUMSTAT p_emp_ = 0.036 from 1000 permutations).

Table 3 shows the detailed results for each gene in the subgroup of secreted factors, which showed the strongest association. In 7 of the 10 genes, SNPs with a p-value below 0.05 were found. Three genes contained only one SNP with a p-value below 0.05. In four genes there were two or more SNPs with a p-value below 0.05, and these genes also contained the two most significant SNPs, found in the *LGALS3* and transforming growth factor beta 2 (*TGFB2*) genes. We tried to replicate the results of this study in the second phase of the GenoMEL GWA study [35], consisting of 1450 melanoma cases and 4047 controls (from Italy, France, Scandinavia, Spain, UK, Netherlands, Poland and Israel), but no evidence of association with the pathway was seen (Table 4).

**Table 3:** Detailed results for the subgroup of secreted immunosuppressive factors.

GeneSymbol	# SNPs	# SNPs p≤0.05	min. p-value
IDO	26	6	0.00128
IL10	21	1	0.03905
TGFB1	20	1	0.00141
TGFB2	28	2	0.00083
CCL17/CCL22	31	0	0.10590
IL12A	27	0	0.05448
IL12B	20	0	0.07014
IL17A	32	1	0.00631
LGALS1	24	4	0.00565
LGALS3	11	8	0.00033

Number of SNPs in the gene region, number of SNPs with a p-value below 0.05 and the minimal p-value of the SNPs (logistic regression analysis) in the gene region are shown.

**Table 4:** Attempt to replicate the results of the pathway analysis in the second phase of data from the GenoMEL GWA study.

Set	SUMSTAT	SUMSQ
All genes	0.167	0.280
Anergy	0.001	0.002
Costim.	0.291	0.411
Treg	0.690	0.794
Secreted	0.738	0.783
tDC	0.701	0.761

1450 melanoma cases and 4047 controls were included in the analysis. Empirical p-values established by 1000 in-cluster (GenoMEL regional groups: Israel, Italy, France, Poland, Scandinavia, Spain, UK/Netherlands) label permutations are shown for the pathway statistics SUMSTAT and SUMSQ.

## Discussion

Using pathway-based analysis, preliminary evidence for an association between genes involved in immunosuppression and melanoma risk is provided by this study. The pathway itself and the genes to include within it were chosen completely blind to the results of the GenoMEL study, yet the observed level of evidence for association was only seen 10 times in 5,000 permutations. The approach applied here uses the most significant SNP within each gene to form the pathway statistic, as suggested by Wang and colleagues [25]. Instead of using a weighted Kolmogorov-Smirnov-like running-sum statistic (used in the original gene set enrichment analysis for genome-wide gene expression profiling) [36], SUMSTAT and SUMSQ statistics were used as suggested by Tintle and colleagues [32]. Both statistics show comparable results, although the SUMSQ statistic tends to have larger p-values in our analyses.

For genome-wide expression analysis, it was found that these statistics, together with label permutations, might lead to many significant gene sets [37]. This may be because very many genes are expressed differentially between the groups being compared, so that many pathway-based gene sets will include at least one differentially-expressed gene, even though the pathway itself is not important. This is less likely in a GWA context, but false positive results may arise due to population stratification. Several steps have been performed here to prevent or rule out spurious associations. Within-cluster permutations were used to preserve the geographical structure of the GWA study in the permuted data sets. In addition to the geographical region of the respective GenoMEL groups, an adjustment for the first three principal components (PCs) was performed in the logistic regression analysis to further reduce the potential effect of population stratification (Table 1 in Information S1). Although slightly less significant empirical p-values were achieved by this method, the results remained stable. Furthermore, 100 random gene sets were analysed by the same method (Table 5 in Information S1). Only 10 of 100 random gene set showed a nominally significant result suggest a type 1 error rate of 0.10 (95% CI 0.05, 0.18). A slightly increased type 1 error rate might be explained by the fact that some of the 100 random gene sets are likely to include a gene associated with susceptibility for melanoma. Assuming 0.12% of genes to be associated with melanoma risk (approximately 24 genes of 20,000 genes in the genome), the probability that at least one of these is included in a random gene set (including 43 genes) is 5%, leading to some gene sets showing inflated evidence of association. For instance, random gene set number 81 (Table 5 in Information S1) contains the gene CDK10, which is found in a region of genome-wide significance in melanoma association studies [21].

As pointed out by Wang et al. [25], the use of the most significant SNP within each gene is only one possibility. This approach could be strongly influenced by a few highly significant SNPs (occurring by chance in the GWA study) being present within the gene set. In a recent study, the second most strongly associated SNP in each gene was used to reduce the chance of this [38]. An alternative approach to pathway analysis, comparable to the approach of Holmans et al. [27], is provided by the gene set test within PLINK. Instead of using one SNP per gene, this approach uses a predefined p-value threshold for the inclusion of SNPs from the initial association analysis into the pathway-based analysis. It further removes SNPs in linkage disequilibrium (LD) based on a predefined criterion. The mean p-value of the selected SNPs is then used as the summary pathway measure, and significance is assessed by case-control label permutation as above. As a secondary analysis, we also applied this method to the data, but found little evidence of association (Table 3 and 4 in Information S1). The results might be expected to be sensitive to selection of both parameters (SNP cut-off p-value and LD criterion), although no differences are found in this study for different p-value thresholds (Table 3 in Information S1). Similarly no difference in the conclusions for the complete gene set was detected by changing the R^2^ filter criterion to 0.8 (data not shown). This approach takes all predefined significant SNPs into account but makes no use of the gene level, which is used in the main analysis presented here. The lack of association may be the result of the introduction of too much noise from genes with large numbers of SNPs. Recently, a comparison of different pathway-based analysis methods including the PLINK gene set test as well as the approaches of Holmans et al. [27] and Wang et al., [25] was performed [39]. The simulation studies suggest that the PLINK gene set test has higher power than the two other approaches. However, the methodologies for pathway-based analysis applied to SNP data sets are still under development, and further simulation studies with a broad range of scenarios have to be conducted before firm conclusions can be drawn.

If validated, the results from this study give an interesting insight into the biology of melanoma susceptibility. The subgroup of secreted factors contains several molecules which are crucial in the crosstalk between tumour cells and the host immune system. The *IDO1* gene encodes an enzyme crucial for the tryptophan catabolism and promoting the arrest of T lymphocyte proliferation by tryptophan deprivation [40], [41]. Non-synonymous coding gene variants in the *IDO1* gene have been associated with an altered gene expression, [42] and it would be of interest to analyse the LD between these coding variants and the SNPs showing association in this analysis. The two Galectins (*LGALS1* and *LGALS3*) are associated with the survival of effector T lymphocytes and may change the balance of the immune response towards an anti-inflammatory cytokine profile [43]. It has been shown that *LGALS3* is regulated by the microphthalmia-associated transcription factor (MITF), which has a pivotal role in melanocyte development and melanoma [44]. Moreover, the serum level of Galectin-3 has been significantly associated with the prognosis of the melanoma patients [45]. The role of these galectins and respective gene variations in melanoma susceptibility has to be further evaluated. TGFB1 and TGFB2 are key immunosuppressive cytokines [46]. Currently, only variations in the *TGFB1* gene have been analysed with regards to the risk of melanoma with conflicting results [18], [19]. This study provides some evidence that variants in *TGFB2* might also be associated with melanoma susceptibility.

Further analyses are needed to confirm the results of this study. No evidence of association with the overall pathway was seen when applied to the second phase of the GenoMEL study (Table 4) [35]. There are several possible reasons for this lack of replication. First, it is likely that the immunosuppression pathway is not among the strongest predictors of melanoma risk (which are related to nevus development and skin pigmentation) and that the gene set selected includes some genes not associated with susceptibility, resulting in low power to detect association even at the pathway level. Secondly, it could be that initial result is a statistical false positive. Our analysis could be likened to examining a candidate SNP for association with disease. In each case, although we have only examined one hypothesis, motivated by biological understanding, p-values of this magnitude (0.001) can arise by chance. Thirdly, the lack of replication could be due to heterogeneity between the two phases of the GenoMEL study, which differed slightly both in the distribution of geographical region of origin and of case ascertainment criteria (see Information S1 for further description). Looking at the results in more detail, the three SNPs showing the highest association in the first analysis showed no evidence of association in the second, but this was not readily explained by differences in geography or reason for ascertainment, although other sources of heterogeneity (e.g. site of melanoma) may exist. This is one of the first pathway-based analyses in which an attempt to replicate the results at the pathway level has been reported. The lack of replication is disappointing but may presage more general difficulties in replicating results for complex hypotheses, that may be susceptible to the effects of heterogeneity and low power. We hope others using these methods will be encouraged to attempt to replicate their own results.

In conclusion, the results presented here suggest that variants in the gene set of immuno-suppressive factors, and especially in the subgroup of secreted factors, may be associated with the susceptibility to melanoma. Although the methodology has to be further evaluated and developed, and we have so far not replicated these results, this study underlines the potential of pathway-based methods for complementary analyses of GWA data sets.

## Supporting Information

Information S1 **Contains additional detail on comparisons between Phase 1 and Phase 2 of the GenoMEL GWA study, supplementary methods and further tables of results.**
(DOCX)
