# Supplementary material for: Pathway-Based Analysis of a Melanoma Genome-Wide Association Study: Analysis of Genes Related to Tumour-Immunosuppression
Source: PLoS One. 2011 Dec 27;6(12):e29451. doi: 10.1371/journal.pone.0029451 (PMC3246481; doi:10.1371/journal.pone.0029451)
Supplement: Information S1 — Contains additional detail on comparisons between Phase 1 and Phase 2 of the GenoMEL GWA study, supplementary methods and further tables of results. (DOCX) [file pone.0029451.s001.docx]

**Supporting Information S1**

**Pathway-based analysis of a melanoma genome-wide association study: Analysis of genes related to tumour-immunosuppression**

**Comparisons between Phase 1 and Phase 2 of the GenoMEL GWA study**

Phase 1 consists of cases and controls from France, UK, Netherlands, Italy, Spain, Sweden and Australia, whereas Phase 2 contains cases and controls from France, UK, Netherlands, Italy, Spain, Sweden, Norway, Poland and Israel. The size of the two studies is similar (1539 cases and 3917 controls in Phase 1; 1411 cases and 3928 controls in Phase 2). More details of the two phases of the study are given in Bishop et al, 2009 [1] and Barrett et al, 2011 [2]. In an attempt to better understand the lack of replication, we examined in more detail two of the genes in the pathway that showed the strongest association in Phase 1 (*LGALS1* and *LGALS3*). In *LGALS1*, 4 SNPs from 24 were significantly associated at p < 0.05, with a minimum p-value of 0.0056 for rs4264658, and in *LGALS3* 8 SNPs from 11 were significantly associated, minimum p-value 0.0003 for rs873061. The allele frequencies for these SNPs did not differ between Phase 1 and Phase 2 (rs4264658 minor allele frequency (MAF) 0.23 and 0.22 in Phases 1 and 2 respectively; rs873061 MAF 0.28 in both phases), and there was no evidence of departure from Hardy-Weinberg Equilibrium at the 5% level. In Phase 1 the estimated odds ratios (ORs) (95% confidence intervals) were 1.15 (1.04, 1.28) for rs4264658 and 0.84 (0.76, 0.92) for rs873061, and there was no evidence of heterogeneity of effect for these SNPs between centres (p=0.45 and p=0.14 respectively). However in Phase 2 there was no evidence for association with either SNP (OR 0.94 and 1.05 respectively), with again no evidence of heterogeneity by centre (p=0.61 and p=0.58 respectively). Case ascertainment differs slightly between the two cohorts. In both phases, an attempt was made to preferentially include cases with a family history of melanoma (FH), multiple primary melanomas (MP) or young age at disease onset. Similar proportions of cases had a FH (43% in Phase1, 44% in Phase 2). However there were fewer cases in Phase 2 with MP and no FH (31% in Phase 1 compared with 23% in Phase 2), and more with early onset but no FH or MP (26% in Phase 1, 33% in Phase 2). rs4264658 showed the strongest effect in cases with a FH or MP (OR 1.22 and 1.21 respectively, compared with OR 1.06 for those with young age at onset but no FH or MP). However restricting the analysis of Phase 2 to these subsets, there was no evidence of a similar effect (OR 0.99 and OR 1.02 respectively in cases with these phenotypes). For the top SNP in *LGALS3*, similar effect sizes were observed in all case groups in Phase 1, but not significantly replicated in Phase 2. We are unable to identify any differences between the two cohorts that explain the lack of replication, and our conclusion is that this is likely to be either a false positive result in Phase 1 or a false negative in Phase 2. Phase 2 is of a similar size to Phase1, and thus should have the same power to detect this pathway association as Phase 1, given that this was a candidate pathway, and thus not subject to the bias known as “winner’s curse”.

**Supplemental Methods**

In an additional analysis the first three principal components (PCs) were included as a covariate into the logistic regression (Table S1). The principal components analysis (PCA) was conducted for the original GWA study to account for population stratification [1]. Briefly, on a defined subset of SNPs (thinned to reduce linkage disequilibrium (LD) and applying stringent quality control) PCA was carried out using EIGENSTRAT [3]. Using the first three PCs, it was possible to separate the European samples by geographical latitude, longitude and between a northwest-southeast axis and a northeast-southwest axis, capturing overall 77% of the variation in the first 20 PCs. The rest of the pathway analysis was conducted as described in the Materials and Methods section.

In a secondary approach, the gene set-based test of the software package PLINK was used (Tables S3 and S4) [4]. In brief, the LD between all SNPs is estimated by PLINK first. Second, for each SNP the association with susceptibility to melanoma is tested using a logistic regression analysis with the GenoMEL regional group as covariate. Third, all SNPs with a p-value threshold (0.05, 0.01, 0.001) are selected beginning with the lowest p-value. SNPs in LD with selected SNPs above a thresh­old (R^2^ > 0.1) are removed before further selection of SNPs. Fourth, a mean p-value of the selected SNPs is calculated. Fifth, 100 case-control label permutations of the set of SNPs is performed. For each permutation, steps two to four are repeated. Finally, an empirical p-value is calculated by the number of times the permuted gene set statistic exceeds the original gene-set test statistics of step two divided by the number of permutations.

**Table S1.** Pathway analysis including additional adjustment for principal components 1 to 3 in the logistic regression analysis. Empirical p-values established by 1000 in-cluster (GenoMEL regional group) label permutations are shown for the pathway statistics SUMSTAT and SUMSQ. Nominally significant results are shown in italics.

| Set | SUMSTAT | SUMSQ |
| --- | --- | --- |
| All genes | *0.006* | *0.014* |
| Anergy | 0.166 | 0.194 |
| Costim. | 0.277 | 0.405 |
| Treg | 0.108 | 0.103 |
| Secreted | *0.002* | *0.001* |
| tDC | *0.013* | *0.020* |

**Table S2.** Pathway analysis where the observed data set was replaced by a case-control label permuted data set. Empirical p-values established by 1000 in-cluster (GenoMEL regional group) label permutations are shown for the pathway statistics SUMSTAT and SUMSQ. Nominally significant results are shown in italics.

| Set | SUMSTAT | SUMSQ |
| --- | --- | --- |
| All genes | 0.103 | 0.093 |
| Anergy | *0.036* | *0.024* |
| Costim. | 0.165 | 0.162 |
| Treg | 0.169 | 0.185 |
| Secreted | 0.779 | 0.770 |
| tDC | 0.694 | 0.726 |

**Table S3.** PLINK gene set test for all genes included in the analysis. The analysis was conducted using a LD criterion of R^2^> 0.1 and the three p-value thresholds 0.05, 0.01 and 0.001. The number of significant SNPs, the number of significant SNPs after removal of SNPs in LD and the empirical p-value tested by 100 label permutations are shown.

| Cut-off p-value for SNPs | # sig. SNPs | # sig. SNPs –LD | emp. p |
| --- | --- | --- | --- |
| 0.05 | 80 | 48 | 0.47 |
| 0.01 | 17 | 11 | 0.17 |
| 0.001 | 4 | 2 | 0.33 |

**Table S4.** PLINK gene set test for gene subgroups. A cut-oﬀ p-value of 0.05 and an LD criterion of R^2^> 0.1 were used. The number of SNPs in the subgroups, the number of significant SNPs, the number of significant SNPs after removal of SNPs in LD and the empirical p-value tested by 100 label permutations are shown. Nominally significant results are shown in italics.

| Subgroup | # SNPs | # sig. SNPs | # sig. SNPs –LD | emp. p |
| --- | --- | --- | --- | --- |
| Anergy | 69 | 10 | 5 | 0.37 |
| Costim. | 377 | 21 | 16 | 0.70 |
| Treg | 604 | 31 | 19 | 0.15 |
| Secreted | 240 | 23 | 10 | *0.02* |
| tDC | 402 | 30 | 17 | 0.14 |

**Table S5.** Pathway analysis for 100 randomly sampled sets of genes. Empirical p-values established by 1000 in-cluster (GenoMEL regional group) label permutations are shown for the pathway statistics SUMSTAT and SUMSQ. Nominally significant results are shown in bold italics.

| Random gene set | Number of genes | SUMSTAT | SUMSQ |
| --- | --- | --- | --- |
| 1 | 39 | 0.430 | 0.475 |
| 2 | 39 | 0.600 | 0.618 |
| 3 | 40 | ***0.023*** | ***0.018*** |
| 4 | 41 | 0.424 | 0.368 |
| 5 | 40 | 0.521 | 0.588 |
| 6 | 41 | 0.883 | 0.769 |
| 7 | 39 | 0.580 | 0.543 |
| Continued on Next Page . . . | | | |

| Table S5 – Continued | | | |
| --- | --- | --- | --- |
| Random gene set | Number of genes | SUMSTAT | SUMSQ |
| 8 | 38 | ***0.024*** | ***0.019*** |
| 9 | 35 | 0.262 | 0.242 |
| 10 | 36 | 0.252 | 0.275 |
| 11 | 35 | 0.216 | 0.179 |
| 12 | 40 | 0.414 | 0.329 |
| 13 | 40 | 0.292 | 0.263 |
| 14 | 36 | 0.301 | 0.407 |
| 15 | 36 | 0.417 | 0.500 |
| 16 | 39 | 0.682 | 0.765 |
| 17 | 37 | 0.263 | 0.316 |
| 18 | 35 | 0.225 | 0.250 |
| 19 | 41 | 0.173 | 0.168 |
| 20 | 36 | 0.666 | 0.679 |
| 21 | 37 | 0.254 | 0.277 |
| 22 | 37 | 0.922 | 0.896 |
| 23 | 38 | 0.311 | 0.285 |
| 24 | 38 | 0.441 | 0.581 |
| 25 | 38 | 0.324 | 0.236 |
| 26 | 38 | 0.483 | 0.642 |
| 27 | 38 | 0.592 | 0.527 |
| 28 | 39 | 0.798 | 0.835 |
| 29 | 38 | 0.333 | 0.333 |
| 30 | 37 | 0.418 | 0.439 |
| 31 | 36 | 0.783 | 0.691 |
| 32 | 39 | 0.966 | 0.963 |
| 33 | 37 | 0.573 | 0.667 |
| 34 | 39 | 0.527 | 0.451 |
| 35 | 38 | 0.492 | 0.291 |
| 36 | 39 | 0.367 | 0.423 |
| 37 | 37 | 0.973 | 0.958 |
| 38 | 40 | 0.181 | 0.167 |
| 39 | 36 | 0.238 | 0.232 |
| 40 | 40 | 0.527 | 0.473 |
| 41 | 37 | 0.428 | 0.442 |
| 42 | 41 | 0.278 | 0.309 |
| 43 | 39 | 0.173 | 0.188 |
| 44 | 41 | 0.111 | 0.069 |
| 45 | 41 | 0.772 | 0.792 |
| 46 | 38 | 0.965 | 0.983 |
| 47 | 38 | 0.529 | 0.595 |
| 48 | 41 | 0.678 | 0.547 |
| 49 | 42 | 0.777 | 0.760 |
| 50 | 42 | 0.752 | 0.804 |
| 51 | 37 | 0.646 | 0.697 |
| 52 | 38 | 0.309 | 0.165 |
| 53 | 41 | 0.826 | 0.796 |
| 54 | 36 | 0.111 | 0.071 |
| Continued on Next Page . . . | | | |
| Table S5 – Continued | | | |
| Random gene set | Number of genes | SUMSTAT | SUMSQ |
| 55 | 38 | 0.651 | 0.635 |
| 56 | 37 | 0.266 | 0.114 |
| 57 | 39 | 0.120 | 0.153 |
| 58 | 41 | 0.610 | 0.614 |
| 59 | 39 | 0.266 | 0.238 |
| 60 | 40 | ***0.019*** | 0.064 |
| 61 | 40 | 0.531 | 0.645 |
| 62 | 39 | 0.768 | 0.550 |
| 63 | 36 | ***0.026*** | ***0.019*** |
| 64 | 38 | ***0.028*** | 0.070 |
| 65 | 38 | 0.809 | 0.756 |
| 66 | 38 | 0.204 | 0.213 |
| 67 | 36 | ***0.023*** | ***0.020*** |
| 68 | 35 | 0.358 | 0.275 |
| 69 | 39 | ***0.034*** | ***0.017*** |
| 70 | 38 | 0.896 | 0.799 |
| 71 | 36 | 0.479 | 0.581 |
| 72 | 35 | 0.276 | 0.197 |
| 73 | 36 | 0.757 | 0.519 |
| 74 | 36 | 0.399 | 0.241 |
| 75 | 39 | 0.737 | 0.763 |
| 76 | 41 | 0.143 | ***0.045*** |
| 77 | 38 | 0.938 | 0.943 |
| 78 | 39 | 0.289 | 0.231 |
| 79 | 39 | ***0.005*** | ***0.003*** |
| 80 | 40 | 0.585 | 0.551 |
| 81 | 37 | 0.054 | ***0.000*** |
| 82 | 36 | 0.478 | 0.505 |
| 83 | 38 | 0.296 | 0.360 |
| 84 | 39 | 0.907 | 0.821 |
| 85 | 39 | 0.522 | 0.474 |
| 86 | 39 | 0.464 | 0.404 |
| 87 | 39 | 0.156 | 0.140 |
| 88 | 40 | 0.123 | 0.107 |
| 89 | 40 | 0.312 | 0.277 |
| 90 | 35 | 0.519 | 0.230 |
| 91 | 37 | 0.195 | 0.159 |
| 92 | 36 | 0.781 | 0.581 |
| 93 | 42 | 0.696 | 0.766 |
| 94 | 40 | 0.683 | 0.735 |
| 95 | 35 | 0.058 | 0.139 |
| 96 | 41 | 0.948 | 0.937 |
| 97 | 39 | 0.821 | 0.861 |
| 98 | 37 | ***0.031*** | ***0.038*** |
| 99 | 40 | 0.478 | 0.322 |
| 100 | 37 | ***0.021*** | ***0.023*** |

**References**

1. Bishop DT, Demenais F, Iles MM, Harland M, Taylor JC, et al. (2009) Genome-wide association study identifies three loci associated with melanoma risk. Nat Genet 41: 920-5.
2. Barrett JH, Iles MM, Harland M, Taylor JC, Aitken JF, et al. (2011) Genome-wide association study identifies three new melanoma susceptibility loci. Nature Genetics, 2011; 43:1108-13.
3. Price A, Patterson N, Plenge R, Weinblatt M, Shadick N, et al. (2006) Principal components analysis corrects for stratification in genome-wide association studies. Nature genetics 38: 904–909.
4. Purcell S, Neale B, Todd-Brown K, Thomas L, Ferreira MA, et al. (2007) Plink: a tool set for whole-genome association and population-based linkage analyses. Am J Hum Genet 81: 559-575.
